# The Role of Hypoxia-Regulated MicroRNAs (Hypoxamirs) in Tumor-Associated Macrophage Polarization: A Systematic Review

**DOI:** 10.7759/cureus.109827

**Published:** 2026-05-28

**Authors:** Almunthir Altobi, Danhui Heo, Lina Barman, Santiago Ruiz

**Affiliations:** 1 Biotechnology, University of Technology and Applied Sciences, Sur, OMN; 2 Medicine, University of Szeged Albert Szent-Györgyi Medical School, Szeged, HUN; 3 Family Medicine, Sutter Health Graduate Medical Education, California, USA; 4 Internal Medicine, University of Antioquia, Medellin, COL

**Keywords:** angiogenesis, chemoresistance, exosomes, hypoxamirs, immunotherapy, liquid biopsy, m2 polarization, tumor-associated macrophages, tumor hypoxia

## Abstract

Hypoxia within the tumor microenvironment (TME) triggers the exosomal transfer of microRNAs (hypoxamirs) that reprogram tumor-associated macrophages (TAMs) toward a pro-tumorigenic M2 phenotype. This systematic review defines the molecular pathways and clinical consequences of the hypoxamir-TAM axis across solid malignancies to guide therapeutic translation. A systematic search of PubMed, Embase, and Cochrane library was conducted through February 1, 2026. Following PRISMA 2020 guidelines, 17 studies were included for analysis. Methodological quality was evaluated using the standard Office of Health Assessment and Translation (OHAT) tool. Analysis identified two primary regulatory tracks: a signaling axis mediated by targets such as PTEN, IRF1, and PHLPP2, and a metabolic axis driven by miR-210, let-7a, and miR-30c targeting the iron-sulfur cluster assembly protein (ISCU). These coordinated pathways drive an "angiogenic switch," facilitate "metabolic migration," and induce significant resistance to chemotherapies, including gemcitabine and temozolomide. Fourteen studies achieved a high-confidence Tier 1 OHAT rating. The hypoxamir-TAM axis is a fundamental driver of immune evasion and therapeutic failure. Targeting this dual-axis framework offers a viable strategy for restoring anti-tumor immunity, while circulating hypoxamirs represent high-value liquid biopsy biomarkers for real-time TME monitoring.

## Introduction and background

As solid tumors outgrow their local vascular supply, they develop characteristic hypoxic regions defined by profound oxygen gradients [1]. These niches trigger the stabilization of hypoxia-inducible factors (HIFs), which drive transcriptional programs for angiogenesis, metabolic reprogramming, and therapeutic resistance [2]. Within this microenvironment, cancer cells actively reshape the surrounding immune landscape through paracrine signaling [3]. A primary vehicle for this communication is the release of tumor-derived exosomes - extracellular vesicles that transport bioactive cargo, including microRNAs, to recipient cells [4]. The term "hypoxamirs" has emerged to describe this specific class of hypoxia-regulated, exosomal microRNAs that facilitate the rapid adaptation of the tumor stroma to hypoxic stress [5].

Tumor-associated macrophages (TAMs) are the most abundant immune population within the tumor microenvironment (TME) and exhibit significant functional plasticity [6]. They exist along a spectrum ranging from classically activated (M1-like, anti-tumoral) to alternatively activated (M2-like, pro-tumoral) states. Under the influence of hypoxic signaling, TAMs typically skew toward the M2 phenotype, characterized by the expression of markers such as CD206 and arginase-1 (Arg1) and the secretion of immunosuppressive cytokines such as IL-10 [7]. Beyond promoting vascular sprouting and metastatic dissemination, these cells drive profound immunosuppression and therapeutic resistance. While the recruitment of TAMs is well-documented, the specific molecular interaction by which exosomal hypoxamirs drive this phenotypic switch remains a focus of investigation [8].

Despite the identification of individual hypoxamirs, a synthesis that maps these findings into a unified mechanistic framework is currently lacking [9]. The field has evolved rapidly between 2024 and 2026, with emerging evidence challenging the traditional view of TAM polarization as a purely local event [10]. Recent studies have introduced the concepts of "metabolic migration," where hypoxamirs program macrophages to home specifically toward hypoxic zones, and "systemic immunomodulation," where tumor-derived exosomes reprogram immune landscapes in distant lymphoid organs [11].

Despite the recognized influence of hypoxia on the TME, the specific molecular pathways by which hypoxia-regulated microRNAs (hypoxamirs) reprogram TAMs across different solid tumors remain fragmented. To address this critical gap, we pose the following research question: How do hypoxamirs drive the metabolic and signaling polarization of TAMs? By systematically reviewing 17 preclinical studies, this review defines these dual-axis mechanisms and proposes a translational roadmap for positioning hypoxamirs as both therapeutic targets and non-invasive liquid biopsy biomarkers.

## Review

Materials and methods

Protocol Registration and Reporting Standards

This systematic review was prospectively registered with PROSPERO (CRD420251159310). The study was executed in accordance with the Preferred Reporting Items for Systematic Reviews and Meta-Analyses (PRISMA) 2020 guidelines [12]. The formal protocol was finalized and locked prior to the commencement of study selection to minimize post-hoc reporting bias.

Eligibility Criteria

Selection criteria utilized a modified PICOS (Population, Intervention, Comparison, Outcomes, and Study) framework tailored for preclinical research. We included studies using models of solid tumors, including in vitro co-culture systems and in vivo animal models. Eligible interventions consisted of hypoxic conditions induced via direct low-oxygen exposure (typically 1% O₂), hypoxia-mimetic agents, or the application of cancer-derived hypoxic exosomes with verified microRNA upregulation. Comparators were defined as normoxic controls, control exosomes, or targeted microRNA inhibition. Primary outcomes were macrophage polarization, assessed through phenotypic markers (CD206, Arg1, iNOS), and functional assays evaluating migration, angiogenesis, and pro-tumoral secretomes. Only original research providing primary experimental data was included; reviews, case reports, and human observational studies were excluded from the synthesis.

Information Sources and Search Strategy

A systematic search was conducted across PubMed, Embase, and the Cochrane Central Register of Controlled Trials from 2014 through February 1, 2026. The search architecture combined Medical Subject Headings (MeSH) and text-word keywords related to hypoxia, hypoxamirs, exosomal crosstalk, and macrophage polarization. A supplementary "bridge search" was executed in early 2026 to capture evidence published between 2024 and February 2026. This was supplemented by a manual hand-search of the reference lists of included studies.

Study Selection and Data Extraction

Title and abstract screening were performed independently by two reviewers (AA and LB) using Rayyan software. Full-text versions of eligible studies were retrieved and subjected to a second independent assessment against pre-defined inclusion and exclusion criteria. Discrepancies were resolved through deliberative discussion or, when necessary, consultation with a third reviewer (SR). Data were extracted into a standardized digital form capturing study metadata, experimental models (cell lines and animal strains), specific hypoxia parameters (oxygen percentage and duration), microRNA strand specificity (5p vs. 3p), validated molecular targets, and clinical prognostic correlations.

Risk of Bias Assessment

Methodological quality was evaluated using the Office of Health Assessment and Translation (OHAT) Risk of Bias Rating Tool [13]. Each study was assessed across seven domains, including randomization, exposure characterization, and outcome assessment. Domains were rated on a four-point scale ranging from "definitely low risk" to "definitely high risk." Based on these ratings, 14 of the 17 included studies reached a high level of confidence, representing strong internal validity. The remaining three studies showed limitations due to reporting omissions in selection and performance bias, which were conservatively rated as having "some concerns."

Due to the substantial methodological heterogeneity across the included literature, specifically regarding diverse cancer types, varying hypoxia protocols, and differing outcome measures, a quantitative meta-analysis was not performed. All findings are presented descriptively.

Results

Study Selection

**Figure 1:**
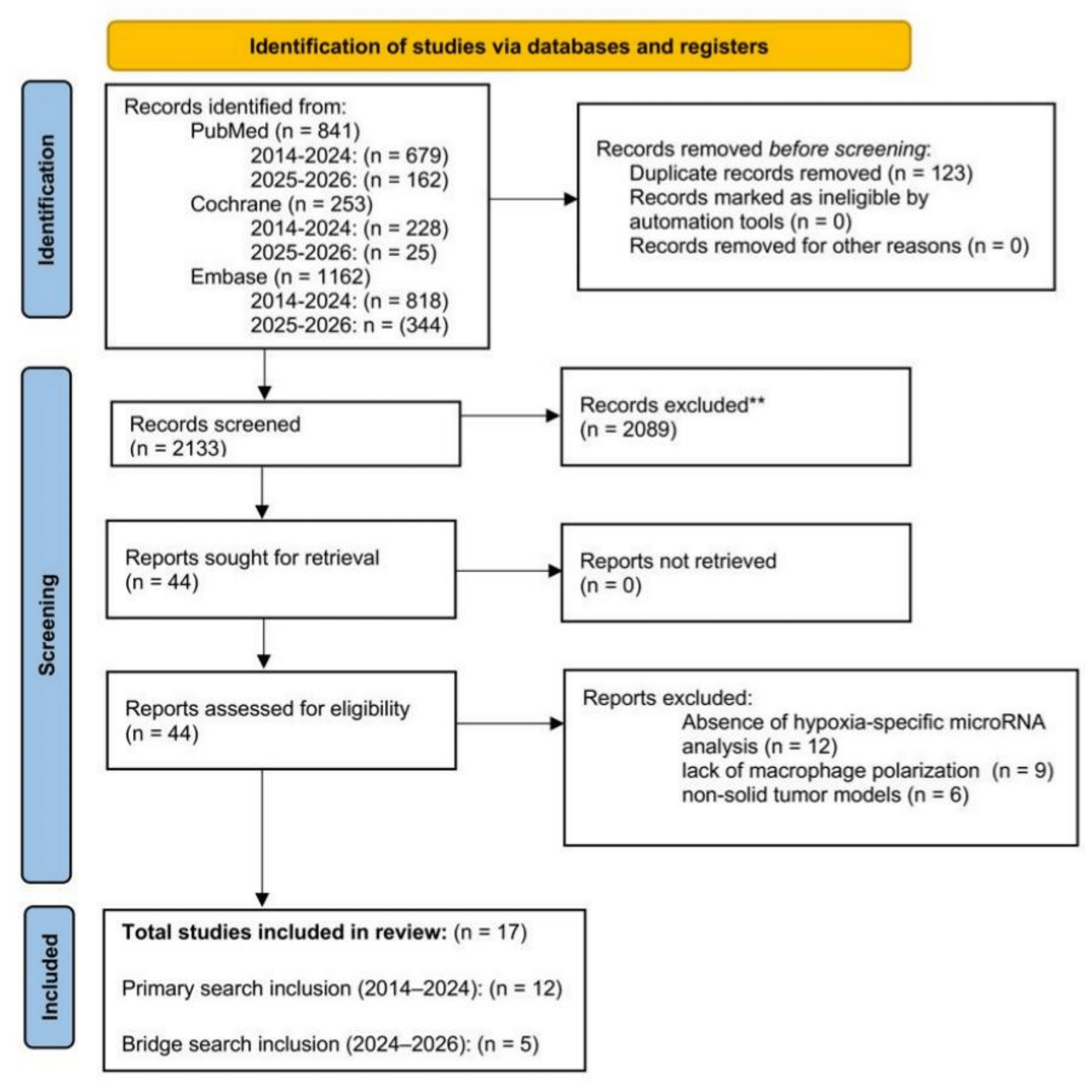
PRISMA 2020 flow diagram of the study selection process PRISMA flowchart template sourced and adapted from Page et al. [12]. The data and screening process represent the authors' original work.

The systematic search identified an initial pool of 2,256 records across PubMed, Embase, and the Cochrane Library. Following the removal of 123 duplicates, 2,133 titles and abstracts were screened. A detailed full-text assessment was performed on 44 records, resulting in the inclusion of 17 studies that met all eligibility criteria for qualitative analysis. Notably, the inclusion of a significant cluster of evidence identified through the 2024-2026 bridge search ensures that the data reflects the most current advancements in the field. Primary reasons for exclusion included the absence of hypoxia-specific microRNA analysis, lack of macrophage polarization data, and the use of experimental models representing hematological malignancies rather than solid tumors. The flow diagram illustrates this systematic identification, screening, and inclusion process (Figure 1).

The 17 included studies were published between 2014 and early 2026, with a significant cluster appearing recently [14,15]. A detailed summary of individual study characteristics, cancer models, and primary molecular findings is presented in Table 1. The evidence base spans eight distinct solid tumor types. Initial investigations focused on pancreatic cancer [14] and colorectal models [15]. Research in lung adenocarcinoma [16] was followed by studies in hepatocellular carcinoma [17], endometrial cancer [18], and additional hepatocellular outcomes [19]. Glioma research was a significant focus [11,20], complemented by investigations in breast cancer [21], ovarian cancer [22,23], further lung cancer mechanisms [24,25], and gastric cancer [26]. The cohort included glioma autophagy analysis [27], nasopharyngeal carcinoma [28], and clinical lung cancer outcomes [29], while broader evidence continues to define these microRNAs as key regulators of oncogenic hallmarks in solid tumors such as colorectal cancer [30].

**Table 1:** Summary characteristics of the 17 included studies This table provides a comprehensive overview of the 17 included studies, detailing the specific cancer types, hypoxia parameters (typically 1% O₂), identified hypoxamir strands, and their primary verified molecular targets. Abbreviations: HCC: Hepatocellular Carcinoma; NSCLC: Non-Small Cell Lung Cancer; TMZ: Temozolomide; OXPHOS: Oxidative Phosphorylation; TME: Tumor Microenvironment; EOC: Epithelial Ovarian Cancer; IRF1: Interferon Regulatory Factor 1

Study (First Author, Year)	Cancer Type	Study Design	Model/Animal Type	Sample Size	Key Hypoxamir	Direct Target/Pathway	Clinical/Prognostic Correlation
Neculachi et al. (2025) [3]	Murine	In vitro	Animal (C57BL/6 cells)	n = 3	miR-210	Anti-inflammatory axis	Pro-survival/tissue repair
Xue et al. (2024) [11]	Glioma	In vitro/in vivo	Animal (C57BL/6 mice)	n = 6 per group	miR-25-3p	PHLPP2/PI3K-AKT-mTOR	TMZ resistance; high-grade recurrence
Guo et al. (2024) [14]	Pancreatic	In vitro/in vivo	Animal (BALB/c nude)	n = 6 per group	miR-210	FGFRL1/PI3K/AKT	Gemcitabine resistance; reduced DFS
Patutina et al. (2026) [15]	Colorectal	In vivo	Animal (BALB/c mice)	n = 5 per group	miR-21, 17, 155	Immune cluster	Systemic tumor suppression
Hsu et al. (2018) [16]	Lung Cancer	In vitro/in vivo	Animal (nude mice)	n = 5 per group	miR-103a	PTEN/AKT & STAT3	Increased microvessel density; poor prognosis
Hu et al. (2024) [17]	HCC	In vitro/in vivo	Animal (C57BL/6 mice)	N Exosomal delivery = 5 per group	miR-21-5p	SP1/XBP1	Increased tumor size; intrahepatic metastasis
Xiao et al. (2020) [18]	Endometrial	In vitro	Human (patient samples/THP-1)	n = 3	miR-21	PTEN (inferred)	Tumor immune escape; reduced T-cell infiltration
Yu et al. (2023) [19]	HCC	In vitro/in vivo	Animal (nude mice)	n = 5 per group	miR-21-5p	RhoB	M2 polarization
Li et al. (2022) [20]	Glioma	In vitro/in vivo	Animal (BALB/c nude)	n = 6 per group	miR-10b-5p	NEDD4L/PI3K/AKT	High WHO grade; poor overall survival (OS)
Park et al. (2019) [21]	Breast / Melanoma	In vitro/In vivo	Animal (C57BL/6 mice)	n = 5 per group	let-7a	Insulin-Akt-mTOR	Metabolic dysregulation; evasion
Tan et al. (2023) [22]	Ovarian Cancer	In vitro/in vivo	Animal (nude mice)	n = 5 per group	miR-1225-5p	TLR2/Wnt/β-catenin	Advanced FIGO stage; lymph node metastasis
Chen et al. (2017) [23]	Ovarian	In vitro	Human (primary cells/lines)	n = 3	miR-940	Exosomal delivery	Ovarian cancer migration
Ma et al. (2023) [24]	Lung (NSCLC)	In vitro	Human (patient cells/cell lines)	n = 3	miR-512-5p	circ_0059665/NOVA2	M2 phenotypic switch
Pontis et al. (2021) [25]	Lung (Risk)	In vitro/in vivo	Animal (nude mice)	n = 6 per group	miR-320	Stromal conversion	Stromal conversion
Yun Zhihua et al. (2019) [26]	Gastric	In vitro/in vivo	Animal (SCID mice)	n = 5 per group	miR-30c	REDD1/mTOR	Metabolic dysregulation; poor prognosis
Xu et al. (2021) [27]	Glioma	In vitro/in vivo	Animal (BALB/c nude)	n = 5 per group	miR-155-3p	IL-6-pSTAT3 feedback	Autophagy-driven migration
Jin et al. (2022) [29]	Lung Cancer	In vitro/in vivo	Animal (nude mice)	n = 5 per group	miR-21	IRF1	Advanced TNM stage; poor overall survival (OS)

Experimental designs relied primarily on in vitro co-culture systems to establish the transfer of exosomal cargo. Analysis evidence base (n=11) incorporated in vivo validation using murine xenograft or orthotopic models [11,14-17,19-22,27,29]. The primary microRNAs central to this crosstalk include miR-210 [3,14], miR-21-5p [17,19,29], miR-155-3p [15,27], and recently characterized strands such as miR-25-3p [11] and miR-103a [16].

Risk of Bias Assessment

**Figure 2:**
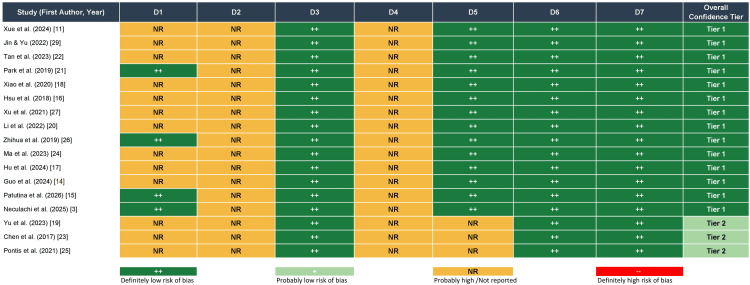
Methodological quality and risk of bias assessment of the included studies Methodological quality assessment of the 17 studies [3,11,14-27,29]. Ratings for each of the seven domains (D1-D7) are provided according to the standard Office of Health Assessment and Translation (OHAT) Risk of Bias Rating Tool. Overall judgment reflects the OHAT tiering system, where Tier 1 indicates high confidence in the body of evidence, and Tier 2 indicates moderate confidence due to "Probably High" or "Not Reported" (NR) risk of bias ratings in one or more domains. Specifically, domains rated as "Probably High" or NR were predominantly due to a lack of explicit reporting regarding the randomization of animals (D1) or the blinding of outcome assessors (D5) in preclinical experiments. Source: Rooney et al. [13]. Abbreviations: D1: Selection Bias (Random sequence generation), D2: Confounding Bias, D3: Performance Bias (Experimental conditions and blinding), D4: Attrition/Exclusion Bias, D5: Detection Bias (Outcome assessment), D6: Selective Reporting Bias, D7: Other Bias OHAT: Office of Health Assessment and Translation

Methodological quality was evaluated using the OHAT tool [13]. Overall, the evidence base is of high integrity, with 14 studies reaching a high level of confidence. Exposure characterization (verification of 1% O_2_ levels) and outcome assessment (standardized M2 marker quantification) were consistently rated as low risk. As visualized in Figure 2, the prevalence of "Unclear" ratings reflects a lack of explicitly reported data on allocation concealment, which is characteristic of preclinical molecular biology rather than a methodological flaw.

Molecular Mechanisms of Hypoxamir-Mediated Polarization

The transition of macrophages from a pro-inflammatory M1 phenotype to a pro-tumorigenic M2 state is driven by two distinct molecular axes. As detailed in Table 1, these mechanisms center on the targeted suppression of specific homeostatic proteins, triggering the activation of oncogenic signaling and a profound reprogramming of cellular bioenergetics.

The signaling axis: AKT and STAT3 Activation: The signaling axis represents the most prevalent regulatory track (n = 10). A central feature is the suppression of phosphatase and tensin homolog (PTEN). When hypoxamirs such as miR-21 and miR-103a are transferred via exosomes, they bind to the 3′-UTR of PTEN mRNA [16,18,20]. This results in the sustained phosphorylation of AKT, which activates transcription factors such as STAT3 [16,18,27]. Activated STAT3 promotes the transcription of markers, including CD206, arginase-1 (Arg1), and IL-10 [25,27]. Other inhibitory nodes, including IRF1 and PHLPP2, are also targeted by miR-21 and miR-25-3p [11,29].

The metabolic axis: Mitochondrial and glycolytic reprogramming: Hypoxamirs orchestrate a shift in macrophage metabolism in 41.2% of the studies (n = 7). The primary mediator is miR-210, which targets the iron-sulfur cluster assembly protein (ISCU) [3,14,16]. This leads to impaired mitochondrial respiration and a glycolytic switch. Evidence suggests these bioenergetic changes contribute to a "metabolic migration" phenotype, where macrophages exhibit enhanced migratory capacity toward hypoxic regions [4,11]. Other hypoxamirs, including let-7a and miR-30c, further modulate this axis via insulin signaling and mitochondrial dynamics [21,26].

Functional and Clinical Outcomes of Macrophage Reprogramming

Enhanced tumor angiogenesis: Twelve of the 17 studies reported an induction of angiogenic activity following polarization [16-19,21-27,29]. In vivo validation models, particularly in glioma and lung adenocarcinoma, demonstrated that co-injecting cancer cells with hypoxamir-programmed macrophages resulted in significantly higher microvessel density (CD31+) and accelerated growth [11,17,20,27].

Systemic and local immune suppression: Hypoxamirs shift the macrophage secretome toward an anti-inflammatory profile (IL-10 and TGF-β). Recent evidence suggests that systemic administration of exosomes enriched with miR-21 and miR-155 can reprogram macrophages in distant lymphoid organs, such as the spleen [15,17].

Therapeutic resistance and clinical prognosis: Hypoxamirs drive resistance to standard chemotherapy in 52.9% of the cohort. Exosomal transfer of miR-210 in pancreatic cancer and miR-25-3p in glioma correlates with reduced sensitivity to gemcitabine and temozolomide (TMZ), respectively [11,14]. High levels of these microRNAs are associated with advanced TNM stages and poor overall survival [19,21,26,29].

Cross-Cancer Generalizability

**Figure 3:**
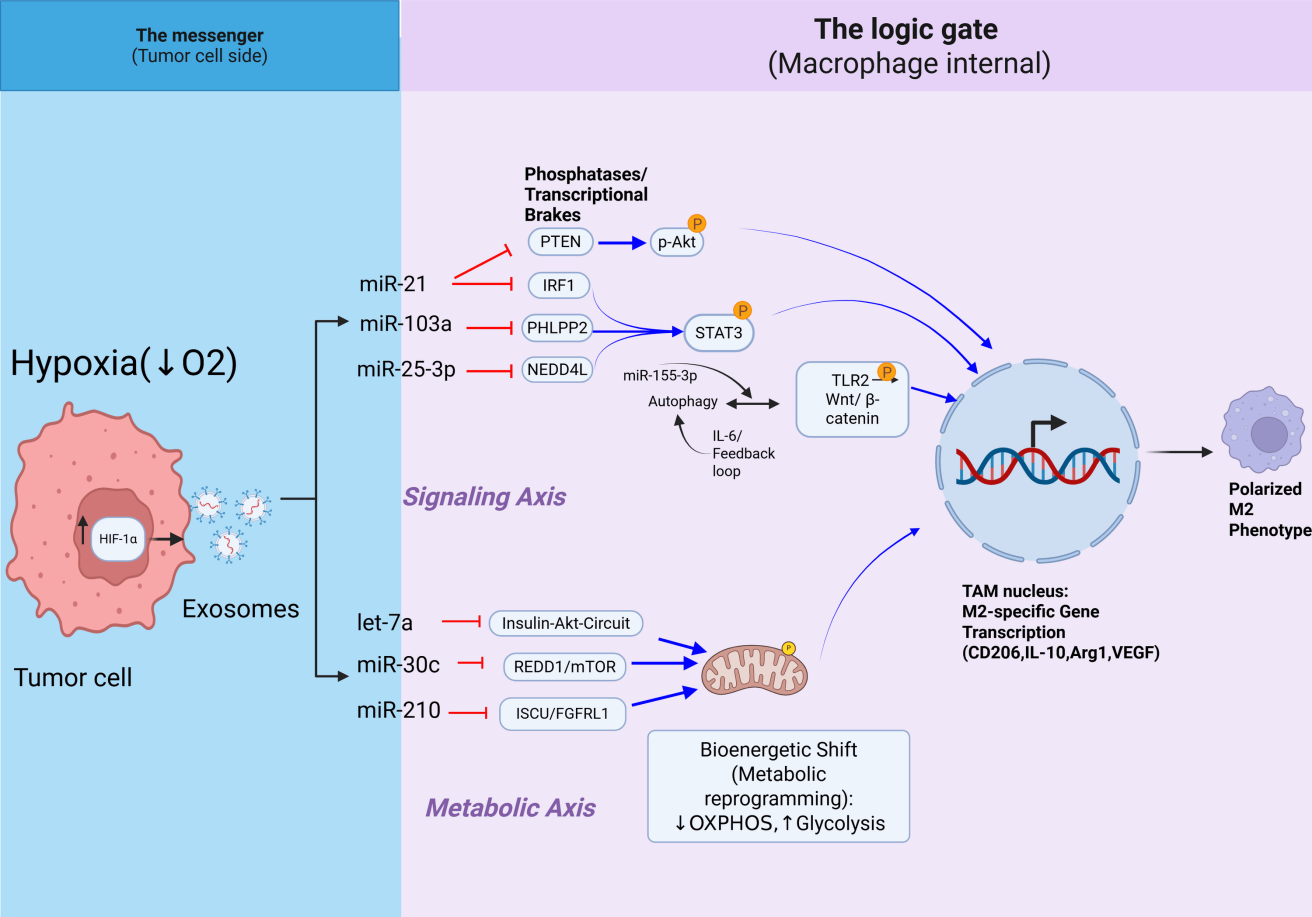
Mechanistic map of the dual-axis regulatory framework for hypoxamir-mediated macrophage polarization This figure illustrates the intracellular "logic gate" within macrophages upon receiving tumor-derived exosomal hypoxamirs. The signaling axis (top) utilizes miR-21, miR-103a, and miR-25-3p to suppress inhibitory proteins, such as PTEN, IRF1, and PHLPP2, leading to the sustained activation of AKT and STAT3. Simultaneously, the metabolic axis (bottom) driven by miR-210, let-7a, and miR-30c targets mitochondrial ISCU and insulin circuits to trigger a profound bioenergetic shift. Together, these tracks converge in the nucleus to drive M2-specific gene transcription (CD206, IL-10, Arg1, and VEGF). Created in BioRender (Science Suite Inc., Ontario, Canada)

The analysis reveals that the regulatory influence of hypoxamirs is largely conserved across diverse solid malignancies. The signaling axis was validated across lung, glioma, endometrial, and hepatocellular carcinoma models [11,16-20,27,29]. Similarly, the metabolic axis was consistently observed in breast, lung, pancreatic, and gastric models [14,16,21,26]. Figure 3 illustrates the distribution of specific hypoxamirs across the eight cancer types, highlighting the dominance of the miR-21 and miR-210 families, which target PTEN and ISCU in six and four of the studies, respectively.

Discussion

Principal Findings

This systematic review analyzes evidence from 17 studies published over a 12-year period to define a dual-axis mechanistic framework for hypoxamir-mediated TAM reprogramming. The signaling axis, validated in 10 of the included studies, utilizes molecules such as miR-21-5p and miR-25-3p to drive the transcriptional changes that establish M2 identity by targeting homeostatic inhibitors such as PTEN and PHLPP2 [11,29]. Simultaneously, the metabolic axis, primarily governed by miR-210, sustains this polarized phenotype through the suppression of ISCU and subsequent bioenergetic remodeling [5]. Our analysis demonstrates that these pathways act in tight coordination: signaling alterations initiate the differentiation toward an M2 state, while metabolic shifts provide the essential bioenergetic requirements to maintain this phenotype and facilitate spatial migration toward hypoxic regions. This mechanistic consensus is grounded in a high-confidence evidence base, where the vast majority of findings originate from studies reaching the highest level of methodological rigor [31,32].

Integration With Broader Literature

The identification of PTEN as a convergent target in six of the 17-study cohort highlights its role as a central node linking hypoxia to immune evasion [33,34]. Furthermore, the inclusion of IRF1 and PHLPP2 as targets in recent studies from the 2024-2026 bridge search adds significant depth to the signaling axis, showing how diverse malignancies utilize distinct molecular "brakes" to achieve similar pro-tumorigenic outcomes. The role of ISCU in metabolic reprogramming, validated in four of the studies, complements the growing field of immunometabolism, suggesting that metabolic states are actively instructive for macrophage function. This review provides a measurable consensus on the role of hypoxamirs in the TME [35-37].

The 2024-2026 Wave: Systemic Effects and Spatiotemporal Dynamics

**Figure 4:**
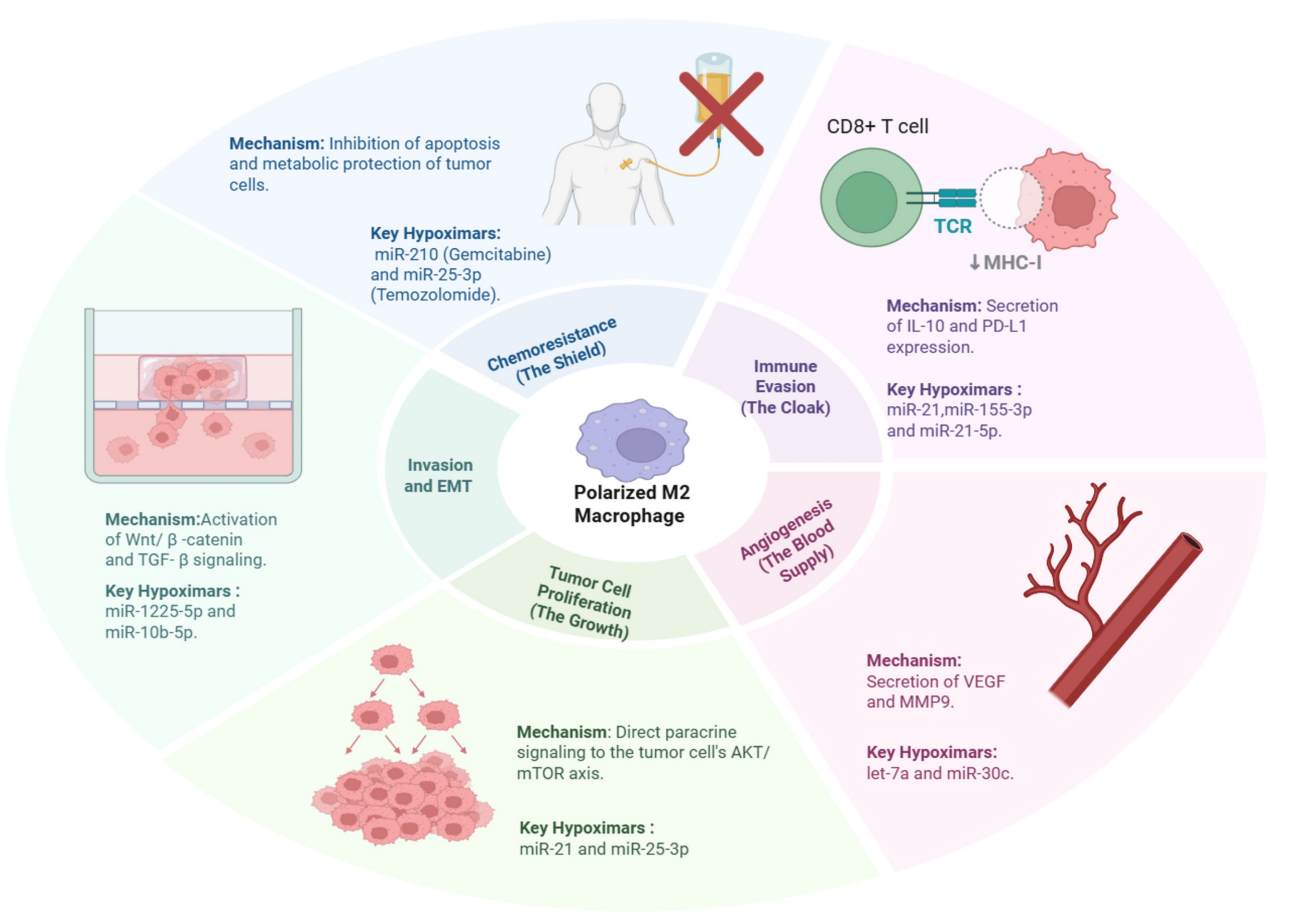
The outcome wheel Functional and clinical outcomes of hypoxamir-mediated macrophage reprogramming This outcome wheel summarizes the diverse pro-tumorigenic effects driven by specific hypoxamirs as identified across the 17-study cohort. Key validated functional outcomes include enhanced tumor angiogenesis (70.6% consensus), local and systemic immune evasion through T-cell suppression, and a 52.9% consensus for heightened resistance to standard chemotherapeutic agents such as gemcitabine and temozolomide (TMZ). The diagram illustrates how individual microRNA strands facilitate tumor cell proliferation, invasion, and the transition toward a metastatic phenotype. Created in BioRender (Science Suite Inc., Ontario, Canada)

A critical insight emerging from the recent 2024-2026 research cluster comprising five of our included studies is that hypoxamir-mediated immunosuppression extends beyond the local TME. Recent findings demonstrate that circulating exosomes can reprogram macrophages in distant lymphoid organs, such as the spleen, suggesting a conserved mechanism for systemic immune dysfunction [17]. Furthermore, the introduction of the "metabolic migration" concept (Figure 4) reveals a spatiotemporal dynamic where miR-210 programs macrophages to home specifically toward severely hypoxic zones. This creates a potent feed-forward loop that concentrates immunosuppressive activity where it is most functionally effective. These advancements, predominantly derived from methodologically robust studies, challenge older models that viewed TAM polarization as a static event and instead highlight a dynamic, spatially organized process driven by continuous exosomal transfer.

Implications for Cancer Immunotherapy

Defining these mechanisms identifies multiple therapeutic strategies. Inhibition of specific hypoxamirs using anti-miR oligonucleotides targeting miR-21 or miR-210 could effectively reverse M2 polarization. Alternatively, blocking exosome biogenesis or uptake could prevent the transfer of pro-tumorigenic cargo, preserving the anti-tumor M1 population. Metabolic modulation, such as the use of glycolysis inhibitors, might disrupt the bioenergetic shift required to sustain the M2 state [38,39]. These approaches could be particularly effective when combined with immune checkpoint inhibitors, as reducing PD-L1-expressing M2 TAMs may overcome resistance to PD-1/PD-L1 blockade. Furthermore, the consensus among nine studies in our cohort linking hypoxamirs to resistance against gemcitabine and temozolomide (TMZ) suggests that targeting this axis could restore sensitivity to standard chemotherapies [40-42].

Methodological Considerations and Study Quality

The OHAT assessment revealed that, while 15 studies in the cohort reached a high level of confidence for exposure and outcome characterization, the reporting of randomization and blinding was frequently insufficient across the evidence base. This remains a common challenge in preclinical molecular biology, where the emphasis often lies on mechanistic discovery rather than strict adherence to reporting standards such as the Animal Research: Reporting of In Vivo Experiments (ARRIVE) guidelines. The prevalence of "not reported" ratings in our risk of bias analysis highlights a critical need for improved transparency in experimental design to enhance the reproducibility of hypoxamir research. Additionally, significant heterogeneity was observed in how M2 polarization was quantified, ranging from flow cytometry for surface markers, such as CD163 and CD206, to secretome analysis of IL-10 and TGF-β, which makes direct quantitative comparisons between studies difficult. Standardized phenotyping panels are needed to allow for more robust analysis in the future [43-46].

Limitations and Future Directions

Several limitations warrant consideration. Publication bias likely skews the evidence base toward positive results, as studies failing to identify significant hypoxamir effects are inherently less likely to be represented in the literature. Furthermore, this review focused exclusively on solid malignancies across eight distinct cancer types, which may limit the direct applicability of these findings to hematological malignancies [47,48]. Methodologically, the frequent reliance on immortalized cell lines such as THP-1 and RAW264. Seven across the 17-study cohort may not fully capture the physiological complexity of primary human macrophage biology [42,49-51]. Additionally, the use of immunocompromised mouse models in many in vivo validation studies prevents a comprehensive assessment of how the hypoxamir-TAM axis interacts with the adaptive immune system in a fully competent host.

**Figure 5:**
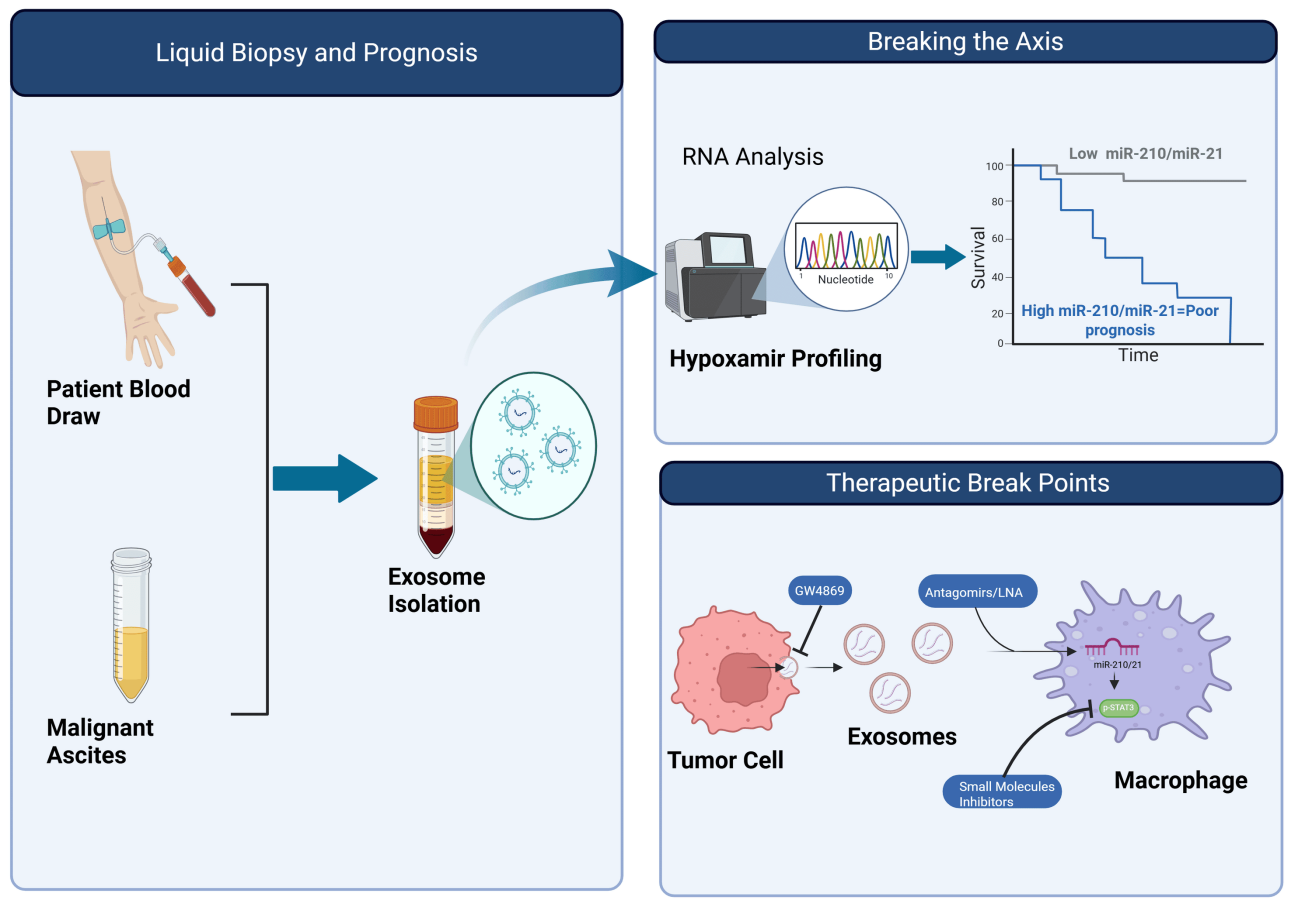
The translational roadmap Translational roadmap and clinical applications of exosomal hypoxamirsThis visual framework identifies the clinical potential of exosomal hypoxamirs as non-invasive liquid biopsy biomarkers for monitoring tumor hypoxia and TAM infiltration. High expression levels of specific hypoxamir strands (e.g., miR-210, miR-21) correlate with advanced disease stages and poor patient survival, as shown in the prognostic survival curves. The diagram further highlights strategic therapeutic "break points," such as the use of exosome secretion inhibitors (GW4869), antagomirs/LNAs, or small molecule signaling inhibitors to disrupt the pro-tumorigenic axis and restore anti-tumor immunity. Abbreviations: LNA: Locked Nucleic Acid; STAT3: Signal Transducer and Activator of Transcription 3 Created in BioRender (Science Suite Inc., Ontario, Canada)

Advancing this field requires a transition from the current high-confidence preclinical foundation toward clinical validation and higher-resolution mapping, as summarized in our translational roadmap (Figure 5). Phase I and II trials are necessary to evaluate the safety and efficacy of hypoxamir-targeted therapies, particularly in combination with immune checkpoint inhibitors. At the bench, spatial profiling and multiplex imaging can map the distribution of exosome uptake and macrophage states within human specimens to verify the "metabolic migration" patterns observed in vitro [52-54]. Integrating single-cell multi-omics will allow researchers to capture the transitional states of macrophages as they navigate the tumor-stroma interface. Future work should also explore the synergy between different hypoxamirs, specifically the combined influence of the miR-21 and miR-210 families, which collectively dominate 10 of the studies in the current literature. Prospective cohort studies are required to correlate circulating hypoxamir levels with tumor hypoxia imaging and clinical outcomes, validating their utility as robust liquid biopsy biomarkers for real-time monitoring of the immune microenvironment [55,56].

## Conclusions

This systematic review establishes a dual-axis framework through which exosomal hypoxamirs orchestrate the functional reprogramming of TAMs. We identify a signaling axis driven by molecules such as miR-21-5p, miR-103a, and miR-25-3p and a metabolic axis mediated by miR-210, let-7a, and miR-30c as the primary regulatory tracks for M2 polarization. These convergent pathways drive critical pro-tumorigenic outcomes, including enhanced angiogenesis, systemic immune suppression, and a heightened resistance to standard chemotherapeutic agents, such as gemcitabine and temozolomide. Emerging data further refine this model by introducing the concepts of "metabolic migration" and systemic immunomodulation, emphasizing that hypoxamir-mediated effects are spatially and temporally dynamic rather than static events.

Clinically, the strong correlation between circulating hypoxamirs and advanced disease stages supports their development as high-value liquid biopsy biomarkers. Ultimately, targeting these molecular messengers offers a viable strategy for overcoming therapeutic resistance and enhancing the efficacy of current immunotherapies in solid malignancies.

## Appendices

Appendix 1

**Table 2:** Detailed systematic search strategy and database architecture This supplementary file provides a comprehensive record of the systematic search conducted across PubMed/MEDLINE, Scopus, and the Cochrane Central Register of Controlled Trials from 2014 through early 2026. It includes the specific Medical Subject Headings (MeSH) and text-word keyword combinations utilized, along with the record counts for each database, the duplicate removal process, and the final unique record tally (n=2,133) to ensure methodological transparency and reproducibility.

Database	Search Strategy (Keywords and MeSH Terms)	Search Date/Period	Number of Records	
PubMed/MEDLINE	(("breast cancer"[MeSH Terms] OR "lung cancer"[MeSH Terms] OR "solid tumors"[MeSH Terms] OR "neoplasms"[MeSH Terms]) AND ("tumor-associated macrophages" OR "TAMs" OR "M2 macrophages") AND ("hypoxia" OR "HIF-1 alpha" OR "hypoxia-regulated microRNAs" OR "hypoxamirs" OR "microRNAs"))	2014/01/01 – 2026/02/01	841	
Cochrane Library	("cancer" OR "solid tumors" OR "neoplasms") AND ("macrophages" OR "tumor-associated macrophages" OR "immune cells" OR "tumor microenvironment" OR "inflammation") AND ("hypoxia" OR "microRNAs" OR "hypoxic conditions" OR "gene regulation" OR "immune modulation")	2014/01/01 – 2026/02/01	253	
Scopus	( ( hypoxamir OR ( hypoxia W/15 mir ) ) AND ( "M2-like Macrophage" OR tam ) AND ( "solid tumor" OR cancer OR Adenocarcinoma ) )	2014/01/01-2026/02/01	1162	
Total Records Identified			2,256	
Duplicates Removed			123	
Total Unique Records Screened			2,133	
